# Auditory affective processing, musicality, and the development of misophonic reactions

**DOI:** 10.3389/fnins.2022.924806

**Published:** 2022-09-23

**Authors:** Solena D. Mednicoff, Sivan Barashy, Destiny Gonzales, Stephen D. Benning, Joel S. Snyder, Erin E. Hannon

**Affiliations:** Department of Psychology, University of Nevada Las Vegas, Las Vegas, NV, United States

**Keywords:** development, misophonia, musicality, sound sensitivity (auditory sensitivity), emotions, autism spectrum disorder, Williams syndrome, misophonic reactions

## Abstract

Misophonia can be characterized both as a condition and as a negative affective experience. Misophonia is described as feeling irritation or disgust in response to hearing certain sounds, such as eating, drinking, gulping, and breathing. Although the earliest misophonic experiences are often described as occurring during childhood, relatively little is known about the developmental pathways that lead to individual variation in these experiences. This literature review discusses evidence of misophonic reactions during childhood and explores the possibility that early heightened sensitivities to both positive and negative sounds, such as to music, might indicate a vulnerability for misophonia and misophonic reactions. We will review when misophonia may develop, how it is distinguished from other auditory conditions (e.g., hyperacusis, phonophobia, or tinnitus), and how it relates to developmental disorders (e.g., autism spectrum disorder or Williams syndrome). Finally, we explore the possibility that children with heightened musicality could be more likely to experience misophonic reactions and develop misophonia.

## Introduction

Misophonia is a newly described and complex auditory condition characterized by aversive reactions to particular sounds and the events that generate those sounds. The term misophonia was not introduced into the published literature until 2001 (Jastreboff and Jastreboff, 2001). Over the past two decades, publications on the topic of misophonia have increased from three articles in 2006 to 36 articles in 2021 (Retrieved from PubMed on September 13, 2022). Though a recent consensus paper classified misophonia as a disorder instead of a condition or syndrome (Swedo et al., 2022), people do frequently experience subclinical reactions to misophonic triggers (Wu et al., 2014; Naylor et al., 2021; Sarigedik and Gulle, 2021).

The goal of this literature review is to explore and examine what is currently known about how misophonic reactions are experienced and how they develop within the general population, not only in clinical populations that seek out treatment. Exploring populations that do not have significant disruption in quality of life and experience less severe misophonic reactions, as well as populations without the disorder, could give clues for understanding and treating people with misophonia; however, this research is largely absent from the literature. Therefore, we examine evidence of misophonic reactions during childhood and explore the possibility that early heightened affective sensitivities to a range of sounds might confer a vulnerability for misophonia. Specifically, we cover when and why misophonic reactions may develop in the general and clinical populations, how misophonia is distinguished from other auditory conditions (e.g., hyperacusis, phonophobia, or tinnitus) and developmental disorders (e.g., autism spectrum disorder or Williams Syndrome), and pose the question of whether a heightened sensitivity or preference for music might predispose a child to experience misophonic reactions, even if these reactions do not rise to the level of clinical impairment. We reviewed the literature in these areas (the development of misophonic reactions, other developmental disorders, and auditory experiences) with the goal of covering fundamental concepts, current gaps within the literature, and potential developments within the field. A limitation of this review is that only research published in English was included.

## Characterization of misophonia

Misophonia is typically characterized by irritation and/or disgust that individuals may experience when hearing certain sounds, such as breathing, drinking, and throat clearing, but especially eating and chewing (Edelstein et al., 2013; Schröder et al., 2013; Wu et al., 2014; Brout et al., 2018). The term misophonia translates directly as the “hatred of sounds.” The sounds that create the negative response in people with misophonia are called “triggers” or “misophonic sounds” (Edelstein et al., 2013, 2020; Brout et al., 2018). Triggers are usually repetitive or periodic in nature, and they create “misophonic responses,” negative emotions that often lead individuals to remove themselves from the environment or sometimes to act with anger and aggression. These responses and reactions and the triggers that elicit them differ across individuals and have varying levels of severity (Edelstein et al., 2013; Brout et al., 2018). Further, misophonia is defined separately from phonophobia (the fear of sounds) and hyperacusis (discomfort or pain due to the intensity, or loudness, of a sound) (Jastreboff and Jastreboff, 2001, 2002; Schröder et al., 2013, 2014; Tyler et al., 2014).

Swedo et al. (2022) codified a consensus definition of misophonia, summarizing 93 definitional statements with 80% or greater expert agreement. This definition highlights the nature of misophonic triggers, the behavioral and negative emotional reactions to these triggers, and expected comorbidities. The consensus definition also separates misophonic reactions from the impairments they may create, which are described in the International Classification of Functioning, Disability, and Health (World Health Organization [WHO], 2001) and will assist clinicians in formally diagnosing misophonia. Within the domain of *body functions*, misophonic reactions may affect *emotional* and *perceptual mental functions*, in which heightened levels of negative emotion and perceptions of sounds are considered aversive instead of neutral. To the extent that misophonic reactions preclude processing of other sounds, they may also impair *sensory bodily functions*. These impairments may interact with the *sound quality* environmental factor of functioning and disability, in which only certain sounds in the environment give rise to misophonic reactions. Misophonic reactions may also affect *participation* in a variety of activities, including impairments in *listening*; *receiving communication*; *handling stress*; and *engaging* in *particular interpersonal relationships*, *education*, and *employment*.

There are a range of self-report questionnaires available to researchers that can be used to determine (1) if someone experiences misophonic reactions and (2) the threshold or degree of severity of the symptoms. A comprehensive list and differences of questionnaires that have and have not been validated can be found in Rinaldi et al. (2021). The questionnaires that have been validated for assessing misophonic reactions are the Misophonia Quotient (MQ; Wu et al., 2014), the Amsterdam Misophonia Scale (A-MISO-S; Schröder et al., 2014; Jager et al., 2020; Naylor et al., 2021; Sarigedik and Gulle, 2021), the Selective Sound Sensitivity Syndrome Scale (S-Five; Vitoratou et al., 2021), the Duke Misophonia Questionnaire (DMQ; Rosenthal et al., 2021), Misophonia Response Scale (Dibb et al., 2021), and MisoQuest (Siepsiak et al., 2020). Though these questionnaires can assess the severity of misophonic reactions, these assessments were created using different definitions of misophonia and need to be evaluated using the consensus definition of misophonia (Swedo et al., 2022).

The self-reported prevalence of individuals who experience misophonic reactions in the general population ranges from 20 to 55% (Wu et al., 2014; Naylor et al., 2021; Rinaldi et al., 2021; Sarigedik and Gulle, 2021). Studies measuring misophonia sensitivity in large (*N*∼500) non-clinical samples of undergraduate college students using the MQ (Wu et al., 2014) and the *Sussex Misophonia Scale* (SMS) (Rinaldi et al., 2021) report that 20–40% of participants have sound sensitivities that significantly affect their lives. Even higher prevalence rates of 50–55% have been observed in studies using the A-MISO-S (Naylor et al., 2021; Sarigedik and Gulle, 2021), with roughly 40% of participants reporting symptoms that are mild, ∼12% moderate, and less than 1% reporting severe symptoms. However, these prevalence estimates are derived from self-reported symptoms and disability, which are typically saturated with negative emotion instead of disorder-specific features (Oltmanns et al., 2018). As a result, these estimates are likely inflated relative to those that would be established through a clinical interview establishing distress and dysfunction specific to misophonia using the new consensus definition (Swedo et al., 2022).

Despite the growing interest in misophonia, it has not been formally recognized as a distinct neurological, audiological, or psychiatric disorder according to either ICD-11 or DSM-5 (-TR) diagnostic criteria (Brout et al., 2018; Lewin et al., 2021). Because of this, the presence and extent of misophonia may be largely underrepresented in the literature (Ferreira et al., 2013; Lewin et al., 2021) or grouped with other dysfunctions of auditory perception with decreased sound tolerance, like hyperacusis (Jastreboff and Jastreboff, 2002, 2013, 2015). Decreased sound tolerance disorders fall under an umbrella of dysfunctions defined by negative reactions to sounds that surpass those that would be expected from an average listener (Jastreboff and Jastreboff, 2002, 2013, 2015). Examples include diplacusis (an anomaly whereby the pitch of a single tone is perceived differently by the two ears; Di Stadio et al., 2018), polyacusis (when more than two tones are perceived from a single sound simultaneously; Jastreboff and Jastreboff, 2015), tinnitus (phantom auditory perception without corresponding activity in the cochlea or external sound source; Jastreboff and Jastreboff, 2001, 2015; Moore, 2012), and most commonly, hyperacusis (Jastreboff and Jastreboff, 2015). Hyperacusis and misophonia have distinct profiles from one another (Jastreboff and Jastreboff, 2015). In hyperacusis, patients’ negative reactions are dependent on the physical characteristics of the sound (e.g., spectrum and intensity/loudness) (for reviews, see Baguley and Hoare, 2018; Potgieter et al., 2020), whereas in misophonia, patients’ negative reactions are dependent on the meaning and context for the individual and typically to specific sound categories (Jastreboff and Jastreboff, 2002, 2013, 2015; Hansen et al., 2021).

As noted by Rinaldi et al. (2021), misophonia assessments typically exhibit poor divergent validity to separate out similar conditions like hyperacusis. Studies that investigate whether someone experiences misophonia or another hearing disorder have to use additional questionnaires or surveys for differential diagnosis (Rouw and Erfanian, 2018; Rinaldi et al., 2021). This is especially important since misophonia and hyperacusis seem to co-occur together with similarly reported symptoms (Jastreboff and Jastreboff, 2013, 2015). Only a few questionnaires have been created and validated to assess hyperacusis across the general population, and at present, no questionnaire differentiates hyperacusis from misophonia (Jastreboff and Jastreboff, 2014; Baguley and Hoare, 2018). Ultimately, further research is needed to clarify the relationship between misophonia and these disorders.

Several other neurological, medical, and psychiatric disorders besides misophonia also entail over-responsivity and sound intolerance. Migraine headaches are a neurological condition characterized by unilateral throbbing headache, photosensitivity, and increased reactivity to other sensory inputs (Sullivan et al., 2014). Autism spectrum disorder (ASD) is a neurodevelopmental disorder that is often accompanied by sound over-responsivities (Ben-Sasson et al., 2009), and Williams Syndrome (WS) is a neurodevelopmental genetic disorder with musicality and sociability as prominent features (Pinheiro et al., 2011; Lense et al., 2014). Schizophrenia is associated with deficits in gating aversive sounds as measured through pre-pulse inhibition of the startle blink reflex (San-Martin et al., 2020), in which the magnitude of the eye blink to a loud and aversive startle probe is typically reduced after a less intense tone (Grillon et al., 1996). In contrast, though one of the criteria for post-traumatic stress disorder is the experience of an exaggerated startle reflex, larger psychophysiologically assessed startle reflex magnitude is not uniquely associated with post-traumatic stress disorder diagnoses (Pole, 2007) and could be associated with other diagnoses instead, like misophonia. The extent to which these disorders–along with such other disorders as obsessive compulsive disorders, personality disorders, and anxiety disorders–are comorbid with misophonia is unclear (Ferreira et al., 2013; Brout et al., 2018; Rouw and Erfanian, 2018; Erfanian et al., 2019).

Though some misophonic sounds can be considered universally annoying, most are not commonly considered to be negative or aversive to the general population (Edelstein et al., 2013; Schröder et al., 2013). As suggested by Edelstein et al. (2020), a given sound (or sounds) can be a misophonic trigger for one person, but not for another person. Misophonic triggers are often human-produced (e.g., eating or breathing sounds) (Schröder et al., 2013), and although many studies report a predominance of human-made triggers, there are both case studies and empirical research of misophonic reactions to a variety of other sounds, such as keyboard or pen tapping, clinking glasses, clock ticking, refrigerator sounds, etc. (Edelstein et al., 2013; Schröder et al., 2013; Jastreboff and Jastreboff, 2014; Taylor, 2017). Although both human-made and non-human-made sounds have been identified as misophonic triggers (Dozier et al., 2017; Hansen et al., 2021), most people with misophonia report at least some human-made triggers, and Jager et al. (2020) argue that misophonia should not be diagnosed for individuals who report exclusively non-human generated triggers.

In neurotypical populations, distinct neural and physiological responses have been observed for people with misophonia relative to controls. For example, individuals with misophonia had heightened skin conductance responses to misophonic sounds, but not when they watched the same stimulus with the sound removed from the visual stimulus (Edelstein et al., 2013; Kumar et al., 2017). This finding indicates that misophonic reactions are largely driven by sound. Additionally, one study (Schröder et al., 2014) reported that individuals with misophonia had diminished N1 auditory event-related potential amplitude to oddball tones compared to controls, but no difference in N1 peak latency. The authors speculated that this finding might indicate auditory and neurobiological abnormalities in misophonia (Schröder et al., 2014).

A few studies have used brain imaging to examine the neural correlates of misophonic reactions. Schröder et al. (2019) investigated neural differences between individuals with and without misophonia using fMRI and electrocardiography. Compared with controls, individuals with misophonia responded to misophonic triggers with higher ratings of anger, disgust, and sadness, increased heart rate, and increased activation of brain areas associated with auditory processing and the salience network. Another study reported that relative to controls, those with misophonia had larger right amygdala volume based on voxel-based morphometry, greater connectivity from their right and left amygdalae to the cerebellum, and increased ventral attention network connectivity to the occipital cortices and fusiform gyri (Eijsker et al., 2021). The authors propose that the enlarged amygdala could be related to heightened emotional responses, and that increased connectivity between the amygdala and cerebellum may drive the reflex-like physiological reactions to misophonic sounds. Because higher ventral attention network connectivity was found with the occipital cortex instead of the auditory cortex, the authors suggest that this heightened connectivity may reflect enhanced capability to respond to visual aspects of misophonic triggers (Eijsker et al., 2021). Lastly, when Kumar et al. (2021) compared individuals with misophonia to controls, they found three main results. First, despite finding no differences in the auditory cortex between both groups, they saw stronger resting state connectivity between both auditory and visual cortices and the orofacial motor area of individuals with misophonia. Second, they found stronger functional connectivity between the auditory cortex and orofacial motor area when general sounds were played, and third, found increased activation in the orofacial motor area for individuals with misophonia when specific triggers were played to both groups (Kumar et al., 2021). Together, these studies provide preliminary evidence that misophonia is associated with distinct physiological and neural responses to misophonic triggers as compared to other sounds (Edelstein et al., 2013; Schröder et al., 2014, 2019; Kumar et al., 2017, 2021; Eijsker et al., 2021). These atypical neurophysiological responses could reflect experience-driven differences or an early predisposition for intense emotional responses to sound, or some combination of the two.

## Development of misophonia

Given that misophonic reactions are experienced by so many people, especially at a sub-clinical level, a critical question is how and when misophonic reactions occur over the course of one’s life. Although a recent consensus between experts agrees that misophonia most likely emerges in childhood, more research is still needed to determine when children are most likely to begin experiencing their earliest misophonic reactions (Swedo et al., 2022). Individuals who experience misophonia typically report symptoms starting from a very young age and/or for as long as they can remember. In a sample of 301 misophonic patients above the age of 18, Rouw and Erfanian (2018) found that 45% of these patients reported onset of misophonic experiences during childhood, 30% during adolescence, and 15% for “as long as I can remember.” In retrospective studies, most individuals reported that their misophonia symptoms emerged during childhood or adolescence (for a review, see Potgieter et al., 2019), with some reported symptoms not emerging until young adulthood (Boyce, 2015; Tunç and Başbuğ, 2017), or emerging at any point throughout the life span (Zhou et al., 2017; Sanchez and Silva, 2018). Thus, there appears to be individual variation in when people retrospectively report their misophonia symptoms emerging.

Studies using self-report measures of misophonia in younger non-clinical populations describe levels of misophonia severity that are comparable to those observed in adults, with more than half of high school students reporting a clinically significant level of misophonia using the A-MISO-S (Sarigedik and Gulle, 2021), and 11% of 10- to 14-year-old children according to an adolescent version of the *Sussex Misophonia Scale for Adolescents* (SMS-A) (Rinaldi et al., 2022). This provides support for the notion that misophonia emerges prior to adulthood.

How misophonia develops is currently unclear (Schröder et al., 2017; Rouw and Erfanian, 2018; Lewin et al., 2021). One study found that 77% of participants self-reported symptoms worsening with age (Rouw and Erfanian, 2018), whereas another study found a negative association between age and misophonia severity (Vitoratou et al., 2021). If symptoms indeed worsen over time, therapies would presumably be needed to prevent this. Treatments like exposure therapy have not been as successful in case studies to treat misophonia due to non-compliance (Hadjipavlou et al., 2008), and studies that have looked at medication alone or counterconditioning are limited to a handful of case studies (Dozier, 2015a,b; McGuire et al., 2015; Tunç and Başbuğ, 2017; Vidal et al., 2017). In comparison, cognitive-behavioral therapy seems to be successful in reducing misophonia in adults (Bernstein et al., 2013; McGuire et al., 2015; Reid et al., 2016; Potgieter et al., 2019; Jager et al., 2021), and perhaps also in younger populations (see Lewin et al., 2021 for a preliminary proof of concept); however, those with a higher severity of misophonia symptoms seem more likely to respond to treatment (Schröder et al., 2017). The effectiveness of cognitive-behavioral therapies in reducing the severity in misophonic symptoms could implicate two mechanisms in the development of misophonia, which await larger-scale treatment dismantling studies to explore. For the first mechanism, if the behavioral components represent the active therapeutic ingredients, such findings would point to a role of learning in misophonia, in which initial hyperarousal in response to a specific sound or sounds might lead to associations between those sounds and aversive emotional and physiological responses (Schröder et al., 2017). For the second, if individuals were to use maladaptive behaviors (such as avoidance) to escape trigger sounds, this would further exacerbate symptoms of misophonia over time, which behavioral interventions would help mitigate (Lewin et al., 2021). Alternatively, if targeting cognitive elaborations and schemas related to misophonic triggers improves patient function, the cognitive components of interventions may provide a means of coping with sensitivities.

In either case, if misophonia is a learned behavior, it is unclear whether or not misophonia could be induced in any person under the right circumstances, as proposed by Rouw and Erfanian (2018), or if certain individuals are predisposed to experience intense emotional responses or aversive reactions to sound. Interestingly, Rouw and Erfanian (2018) found that half of the people with self-reported misophonia in their study experienced autonomous sensory meridian responses (ASMR), whereas Vitoratou et al. (2021) found that 29.2% of their participants from their study self-reported experiencing both. This raises the question of whether these same individuals not only have pronounced negative experiences toward misophonic triggers, but also enhanced positive experiences to other sounds as well (see below). Perhaps there are individual differences in the intensity with which listeners experience emotional responses to sound, and further research attempting to understand these endophenotypes could help address this question of whether or not certain individuals are more or less vulnerable to acquiring misophonia.

## Misophonia, autonomous sensory meridian response, and frisson

Although misophonia is characterized by negative emotional reactions to sounds, an open question is whether those who experience misophonic reactions also have heightened reactions to other sounds–both positive and negative. Autonomous sensory meridian response (ASMR) is a phenomenon typically triggered by everyday stimuli that induce a state of relaxation, positive feelings, and tingling sensations that originate from the head region and spread to the rest of the body (Barratt and Davis, 2015; McGeoch and Rouw, 2020). ASMR has been associated with specific personality traits, with individuals who experience ASMR having higher scores on openness to experience and neuroticism on the Big Five Inventory of personality (Fredborg et al., 2017). Individuals who experience ASMR may watch or listen to ASMR content several times a week to multiple times per day, usually for relaxation or sleep induction (Barratt and Davis, 2015; Barratt et al., 2017; McErlean and Banissy, 2017; Poerio et al., 2018; Kovacevich and Huron, 2019). ASMR is a common experience as seen by the ubiquity of virtual ASMR communities around the world (Liu and Zhou, 2019), and a high percentage of individuals reporting that they experience ASMR, with one study showing 81% of 1,002 participants experienced ASMR (Poerio et al., 2018).

Like misophonia, those who experience ASMR exhibit larger physiological responses to ASMR triggers relative to those who do not experience ASMR, such as a decrease in heart rate and an increase in skin conductance (Poerio et al., 2018). Also like misophonia, most individuals who report having ASMR say that they have experienced it in some form since childhood (Barratt and Davis, 2015). Interestingly, a large proportion of individuals who experience ASMR also self-report experiencing misophonia (Barratt et al., 2017), and they tend to score higher on misophonia scales relative to controls (McErlean and Banissy, 2018).

While misophonia seems to be a type of negative sound sensitivity, ASMR is a type of sound sensitivity that is predominantly perceived as affectively positive. Similarly to misophonic triggers, sounds that give rise to pleasurable ASMR experiences in some people (e.g., someone clicking their tongue, trimming their nails, typing on a keyboard, or chewing ice) do not elicit particularly strong reactions in others (Barratt and Davis, 2015). Rather, the types of sounds that induce ASMR vary depending on the person’s specific sensitivity level and experiences with those sounds, which is also very similar to those who experience misophonia (Pruitt, 2019). In fact, some of the very same sounds that produce positive ASMR reactions in one person can produce completely opposite negative misophonic reactions in another person, especially chewing and other eating sounds (McErlean and Banissy, 2017). Sounds such as chewing or slurping would be considered emotionally neutral by most people, but this sound can either elicit heightened negative reactions in people with misophonia or heightened positive feelings in some who experience ASMR. This is consistent with the notion that misophonia and ASMR might both entail increased auditory emotional responses but with contrasting affective valence (Barratt and Davis, 2015).

Frisson, or musical chills, is yet another well-documented sound-induced emotional phenomenon characterized by positive affect and strong physiological reactions while listening to music, notably shivering, goosebumps, and teary eyes (del Campo and Kehle, 2016; McGeoch and Rouw, 2020). Frisson is seen as an overall pleasant experience, with one study showing that chills were significantly correlated with the perceived pleasantness of the songs (Grewe et al., 2007). Although there are distinct differences between ASMR and frisson, including stimulus triggers, duration, and specific emotional responses, some have proposed that ASMR might be a milder, less intense version of frisson (del Campo and Kehle, 2016; Kovacevich and Huron, 2019). Musical frisson seems to be a more common experience than ASMR, with one of the earliest studies on frisson showing that, out of all 249 participants who responded to their survey, about 78.7% self-reported ever experiencing chills (Goldstein, 1980). Although many people experience frisson, it does not occur regularly and differs greatly between people in amount, duration, and the musical pieces that induce them (Grewe et al., 2007). The experience of musical frisson is associated with brain reward circuitry: regional cerebral blood flow increases in left ventral striatum and dorsomedial midbrain, but decreases in right amygdala, left hippocampus/amygdala, and ventral medial prefrontal cortex (VMPF) (Blood and Zatorre, 2001). These regions of activation in the brain reward circuitry have been associated with euphoria and positive emotions, which is consistent with the positive affective experience of frisson.

Although music-induced frisson has been the focus of prior research, relatively little is currently known about the relationship between misophonia and frisson. Even so, frisson and misophonia have some noteworthy similarities. Like misophonia, frisson experiences are consistent, so even after listening to the same piece of music multiple times, a listener can still experience this strong emotional response (Sloboda, 1991). The experiences of frisson and misophonia both vary widely between individuals: virtually any genre of music can induce frisson in a person depending on their preferences, and misophonic triggers also vary depending on the individual and their life history with a given trigger (Salimpoor et al., 2009; Edelstein et al., 2013). Nevertheless, the acoustic features of misophonia and frisson triggers may differ, since misophonic triggers are often repetitive or periodic in nature (Edelstein et al., 2013; Brout et al., 2018), whereas frisson is often and most notably induced when a novel musical event violates expectations, such as an unexpected harmony or entrance of a new voice, or an intense musical feature like dynamic leaps in loudness (Sloboda, 1991; Grewe et al., 2007; Plazak, 2008; Harrison and Loui, 2014). More research is needed to understand the similarities and differences between experiences of misophonia and musical frisson.

Misophonia, ASMR, and frisson all entail high-level auditory affective processing and depend on specific triggers that vary across individuals. Some listeners may have greater likelihood of experiencing ASMR, misophonia, or frisson because of their greater attention to and sensitivity toward a range of both positive and negative meaningful sounds. Although no papers to date directly compare frisson and misophonia, both phenomena are associated with activity in similar areas of the brain and have similar physiological expressions. As for the differences in brain regions between frisson and misophonia, in response to frisson-inducing classical music, a decrease in cerebral blood flow was seen in the left hippocampus, amygdala, and VMPF with positron emission tomography (Blood and Zatorre, 2001), whereas in response to misophonia trigger videos, an increase in activation was seen in some of these same areas with fMRI (Kumar et al., 2017). The amygdala and VMPF are both areas involved in emotional processing and regulation, implicating emotional circuitry in both of these phenomena (Blood and Zatorre, 2001; Kumar et al., 2017). As the intensity of frisson increases, regional cerebral blood flow increases in regions for motor processes, such as the supplementary motor area and cerebellum (Blood and Zatorre, 2001). Though this may explain muscle tension and relaxation when experiencing musical frisson, the increased connectivity between the amygdala and cerebellum when experiencing misophonia may drive the reflex-like physiological reactions to misophonic sounds (Eijsker et al., 2021). As for similarities between frisson, misophonia, and ASMR, listening to frisson-inducing music, misophonia-inducing sounds, and ASMR videos all caused increases in skin conductance and heart rate (Craig, 2005; Salimpoor et al., 2009, 2011; Edelstein et al., 2013; Poerio et al., 2018; Schröder et al., 2019). Furthermore, an increase in the activation of the insula and anterior cingulate cortex, which are parts of the salience network that detects and selects emotionally important information, is also seen with all three emotional phenomena: frisson (Blood and Zatorre, 2001), misophonia (Kumar et al., 2017; Schröder et al., 2019), and ASMR (Lochte et al., 2018). Both frisson and ASMR can be influenced by expectancy effects, in which observers anticipate that a certain stimulus will result in a sensory experience and are therefore likely to achieve that outcome (Cash et al., 2018). The role of expectation in both ASMR and frisson may be due to increased activity in the reward pathway, such as the left and right nucleus accumbens as well as the medial prefrontal cortex, along with brain regions associated with emotional arousal as noted above (Blood and Zatorre, 2001; Lochte et al., 2018; Valtakari et al., 2019). The similarities in physiological responses between frisson, ASMR, and misophonia suggest that these brain regions may be involved in sound-induced emotional responses, both positive and negative. Because many people who experience ASMR also experience misophonia, and ASMR and frisson both similarly heighten reward pathway activity, it is possible that many people who experience both misophonia and ASMR also experience frisson.

## Developmental disorders with heightened sensitivity to sounds

Given that misophonia falls under the umbrella of decreased sound tolerance disorders and appears to at least share some features with other phenomena that begin in childhood, it may be informative to compare misophonia with other disorders that originate in childhood and entail heightened sensitivity to sounds. For example, Williams Syndrome (WS) and autism spectrum disorder (ASD) are neurodevelopmental disorders that are associated with hyperreactivity to both positive and negative sounds and a high prevalence of decreased sound tolerance, including misophonia.

People with WS exhibit higher rates of hyperacusis, auditory fascinations, and auditory aversions (Levitin et al., 2005; Lense et al., 2013), compared to typically developing individuals. Hyperacusis has been proposed to result from a tendency for heightened arousal in the sympathetic nervous system, leading to fight-or-flight reactions (Blomberg et al., 2006). Because of this tendency, hyperacusis could be seen as a vulnerability for psychopathology (Blomberg et al., 2006). The prevalence of misophonia is not as well known in this population, perhaps because most researchers studying WS would not have become aware of misophonia until recently. Individuals with WS who have been diagnosed with hyperacusis often report aversion (misophonia) or fear (phonophobia) of sounds, rather than a decreased tolerance or pain to the sound’s intensity, as is typical with hyperacusis (Baguley and McFerran, 2011; Jastreboff and Jastreboff, 2014; Silva et al., 2021). In fact, studies have reported a high prevalence of auditory aversions within this population, with as much as 85–95% of people with WS reporting aversions to one or more sounds (Klein et al., 1990; Van Borsel et al., 1997; Levitin et al., 2005). The aversive sounds in this population seem to be contextual, and driven by unique individual experiences, as in misophonia. This suggests that misophonia in WS may have been mistaken for hyperacusis (Jastreboff and Jastreboff, 2014). One solution to distinguish this, as suggested by Baguley and McFerran (2011), would be to administer a validated questionnaire to formally diagnose hyperacusis separately from misophonia, and work toward greater consensus in defining hyperacusis (Glod et al., 2020).

Decreased sound tolerance is also experienced by 50–70% of children or adults with ASD at some point in their lives (Williams et al., 2021a). However, similarly to those with WS, clinical questionnaires vary and do not all address which phenomenologically distinct aspects of decreased sound tolerance that the individual with ASD is most likely to be experiencing: hyperacusis, phonophobia, or misophonia (Williams et al., 2021a,b). This differentiation will be important for future research, as recent meta-analyses state that current and lifetime prevalence of hyperacusis in ASD can be as high as 40 and 60%, respectively (Williams et al., 2021b). No studies to date have assessed the prevalence of misophonia in ASD and separated it from the other forms of decreased sound tolerance. Evidence from clinical samples that happen to have patients comorbid with ASD suggest that for cases of decreased sound tolerance in this population, it could be explained by hyperacusis (Amir et al., 2018), misophonia, or both (Williams et al., 2021b). A few of the regions of the brain implicated in the pathology of ASD include the insula, amygdala, and salience network (Uddin and Menon, 2009; Dziobek et al., 2010; Green et al., 2016), which are also regions implicated in misophonia (Kumar et al., 2017; Schröder et al., 2019). Resting-state connectivity between the salience network and amygdala has been seen in neuroimaging studies of children and adolescents with ASD (Green et al., 2016), as well as in adults who experience misophonia (Schröder et al., 2019; Eijsker et al., 2021). This connectivity has been proposed to be related to the sensory over-responsivity seen in people with ASD in response to auditory and tactile stimuli (Green et al., 2016, 2018). It is possible that these networks underlie auditory sensitivities exhibited in both autism and misophonia.

A striking feature of ASD and WS is that both are characterized by unusually positive responses to musical stimuli (Järvinen et al., 2016). Enhanced processing and heightened affinity toward music are seen in varying degrees across both disorders (Levitin et al., 2005; Lense et al., 2013; Järvinen et al., 2016). For example, though language deficits are common in ASD, pitch and melody discrimination are unimpaired or even enhanced in those with ASD compared to typically developing controls (Bonnel et al., 2003; Heaton, 2003; Jones et al., 2011; O’Connor, 2012; Stanutz et al., 2012). From early in development, individuals with ASD tend to prefer and allocate greater attention to musical stimuli than to speech (Blackstock, 1978; Dawson et al., 1998; Kuhl et al., 2005; O’Connor, 2012). This increased awareness and attention to pitch and music could even contribute to language impairments observed in ASD, particularly if greater attention to other sounds interferes with or alters the trajectory of typical language learning (O’Connor, 2012). Likewise, despite auditory sensitivities and aversions to particular sounds and a range of cognitive deficits, those with WS seem to largely exhibit enhanced musical abilities, hypermusicality and high engagement with music (Levitin and Bellugi, 1998; Don et al., 1999; Lenhoff et al., 2001; Lense et al., 2013), however, that is not true for everyone with WS (Don et al., 1999; Thornton-Wells et al., 2010). It is important to note that there are also key differences between WS and ASD especially regarding degree of sociability (Asada and Itakura, 2012). While individuals with WS are typically extremely social and want to interact with others (Jones et al., 2000) and this sociability may drive an affinity for music and musical activities (Zitzer-Comfort et al., 2007), those with ASD often exhibit wide-ranging deficits in social processing and communication (Phillips et al., 2019). Thus, there may be distinct mechanisms and causes for musical affinity in these populations. It is also unclear in both WS and ASD populations whether individuals who show particular affinity or interest in music also tend to have more auditory aversions. Nevertheless, we review this evidence to point out that in principle, stronger negative and positive reactions to emotionally meaningful sounds can exist within the same population.

## Misophonia and musicality

If certain individuals tend to experience stronger negative and positive emotional responses to sounds, sound sensitivities might be more common in people in the general population who show a greater affinity for music and musical activities. Indeed, professional musicians report more sound-related problems such as tinnitus, hyperacusis, and diplacusis than would be expected based on their age and gender (Kähäri et al., 2003, 2004; Jansen et al., 2009; Zhao et al., 2010; Schink et al., 2014; Jastreboff and Jastreboff, 2015; Di Stadio et al., 2018). Although there are many anecdotal accounts of musicians who experience misophonia (Kuehn, 2015), few studies have directly examined whether musicians are more likely than non-musicians to experience misophonia. In one study, “noise sensitivity” did not differ for musicians and non-musicians (although it did negatively predict music listening and enjoyment) (Kliuchko et al., 2015); however, noise sensitivity as measured was not equivalent to misophonia (Weinstein, 1978). A recent study showed that, compared with non-musicians, self-reported misophonia was in fact lower for musicians who practiced from 1 to 7 h per day (Siepsiak et al., 2020). It is unclear whether this result implies that those with a propensity toward music training are actually less likely to experience misophonia, or if misophonia might interfere with regular music practice. The relationship between music training and sound oversensitivity is further muddied by the fact that professional musicians tend to have more long-term exposure to loud sounds (Jansen et al., 2009; Zhao et al., 2010; Schink et al., 2014), so many of the sound-related problems reported above (e.g., tinnitus, diplacusis) in professional musicians may be driven by low-level damage to the auditory system and not necessarily by high-level aversive reactions to sounds, as seen in misophonia (but see Couth et al., 2020).

It is nevertheless also apparent that musicians differ from non-musicians in how they respond to a range of both musical and non-musical sounds, particularly affective sounds. There is abundant evidence that, compared with non-musicians, musicians exhibit enhanced processing of fundamental musical components such as pitch, melody, timbre, chords, and musical rhythm (Franěk et al., 1991; Pantev et al., 2001; Micheyl et al., 2006; Chen et al., 2008; Brattico et al., 2009; Repp, 2010; Schellenberg and Moreno, 2010; Boh et al., 2011; Rammsayer et al., 2012; Matthews et al., 2016). Musicians outperform non-musicians at recognizing emotion conveyed in music (Castro and Lima, 2014; Kantor-Martynuska and Horabik, 2015; Akkermans et al., 2019; Dahary et al., 2020), they have more consistent, more rapid, and/or more intense experiences of both positive and negative musical emotion as reflected by subjective arousal ratings and physiological responses (Steinbeis et al., 2006; Brattico et al., 2009; Dellacherie et al., 2011; Mikutta et al., 2014; Park et al., 2014), and these affective responses are driven by a distinct set of musical cues such as dissonance, mode (major/minor), and harmony (Schön et al., 2005; James et al., 2008; Midya et al., 2019; Battcock and Schutz, 2021). Even the experience of frisson has been reported more often in musicians than in non-musicians (Sloboda, 1991; but see Grewe et al., 2007). Music training also predicts better performance on non-musical speech and language processing tasks and measures (Wong et al., 2007; Parbery-Clark et al., 2009; Bidelman et al., 2013; Zuk et al., 2013; Coffey et al., 2017). In particular, musicians outperform non-musicians at identifying and responding to emotion in speech (Nilsonne and Sundberg, 1985; Thompson et al., 2004; Lima and Castro, 2011). This suggests that individuals who pursue music training or who become professional musicians have enhanced affective responses to both musical and non-musical sounds.

Most studies of musicians and music training are correlational, so it is unclear if the advantages and disadvantages described above result from extensive practice and training-driven plasticity, or if individuals with greater aptitude or affinity for music are more likely to pursue music training or a career in music (Swaminathan and Schellenberg, 2018). Importantly, formal music training is not necessary for listeners to meaningfully relate to and engage with music. Over the course of development, most adult listeners acquire informal musical expertise through day-to-day exposure that allows them to understand the nuances of music, such as generate expectations about when and what will happen in the music, detect wrong notes, dance and sing in synchrony with others, and respond emotionally to music (Hannon and Trainor, 2007; Corrigall and Trainor, 2010, 2014; Hannon et al., 2018). Although some evidence suggests that musicians and non-musicians attend to different structural features of music while performing the same musical tasks (Midya et al., 2019; Battcock and Schutz, 2021; Nave-Blodgett et al., 2021), this is perhaps not surprising given that individual differences in many aspects of music processing are robustly shaped by listening experience, such as exposure to particular cultural traditions or genres of music (Hannon and Trehub, 2005; Demorest et al., 2008; Honing and Ladinig, 2009; Hannon et al., 2012; Ullal et al., 2014). Musicians may therefore represent a rather narrow segment of the general population, as robust and music-specific behavioral and neural responses to music can also be observed in individuals who have no formal music training (Tervaniemi et al., 2006; Vanden Bosch der Nederlanden et al., 2015; Mednicoff et al., 2018; Boebinger et al., 2021; Jacoby et al., 2021). Thus, focusing on differences between musicians and non-musicians may not be as productive as examining individual differences in musical skill and engagement in the general, non-musician population, especially if we are interested in understanding the development of predispositions toward sound sensitivity.

Recent evidence has called into question the popular assumption that music training and practice are the primary drivers of the hearing-related skills and advantages observed in musically trained individuals. A number of tests are now available to assess musical abilities in the general population, such as the Goldsmith Musical Sophistication Index (Gold-MSI) (Müllensiefen et al., 2014a,b), Musical Ear Test (Wallentin et al., 2010), and Profile of Music Perception Skills (Law and Zentner, 2012), and they have revealed considerable individual variation in musical abilities even among individuals who have never had formal music training. Formal music practice/training appears to be only one of several factors (such as cognitive ability and openness to experience) that predict variation in musical abilities (Swaminathan and Schellenberg, 2018). Twin studies suggest that music practice itself is highly heritable, and that music ability is predicted by genetic relatedness and not by practice (e.g., identical twins who differ in practice nevertheless have similar musical abilities) (Mosing et al., 2014). Recent advances in genetics have even identified specific loci associated with singing and musical pitch processing abilities (Ukkola et al., 2009; Park et al., 2012; Tan et al., 2014).

Musical ability (rather than music training) also predicts non-musical skills. For example, non-musicians with high musical ability exhibit enhanced neural encoding of speech comparable to what has been observed among highly trained musicians (Mankel and Bidelman, 2018), and rhythm perception ability predicts discrimination of speech phonemes even after controlling for music training (Swaminathan and Schellenberg, 2017). Similarly, vocal emotion recognition (but not facial emotion recognition) is predicted by music aptitude after controlling for music training, and untrained individuals with high music aptitude were just as good as trained musicians at identifying vocal emotion (Correia et al., 2020). In one study with children aged 6–9, music ability was correlated with language abilities, IQ, personality (only openness to experience was included), and age, and the relationship between musical and language abilities remained even after controlling for music training (Swaminathan and Schellenberg, 2020). This suggests that basic auditory skills and musical aptitude vary meaningfully in the general adult and child populations, and they predict not only musical ability but also non-musical abilities relevant for speech and emotion processing. Although these predispositions presumably drive certain individuals to pursue music as a hobby or profession, an open question is whether they also predict that certain individuals will have stronger, more intense experiences such as misophonia, ASMR, and frisson. Another possibility, as noted above, is that individuals who experience misophonia may be less drawn toward music and less likely to have pleasurable auditory experiences from an early age, or alternatively, perhaps musical activities reduce misophonic reactions by providing more positive auditory affective experiences or by masking aversive sounds and misophonic experiences (Jager et al., 2020; Siepsiak et al., 2020). Research examining potential links between sound sensitivity and musicality, particularly during childhood, might shed light on individual risk factors for developing misophonia, and they may inform treatments that entail using music as a treatment for misophonia (Dozier, 2015a,b,c; Kuehn, 2015; Potgieter et al., 2019).

## Discussion

In this paper, we reviewed what is currently known about the characterization and development of misophonic reactions, developmental disorders that exhibit heightened sound sensitivity or over-responsivity, and misophonia’s relationship to other auditory emotional experiences, including music. Rather than only examining misophonia in treatment-seeking populations, this review aimed to also explore how misophonic reactions may be experienced within the general population. To do this, we discussed how individual differences in development, auditory experiences, and musical predispositions might drive certain people to have stronger affective responses to sounds and audiovisual stimuli. This could influence the likelihood of individuals experiencing misophonia, ASMR, and frisson over the course of development.

Future research should explore individual variation in high-level auditory and affective processing of sounds, including music, to examine whether musicality might be related to the development of other sound sensitivities. Given that very few to any measures of sound-induced emotional experiences (misophonia, ASMR, chills) have been validated with children nor designed to be child-friendly (Schröder et al., 2013; Roberts et al., 2020; Rinaldi et al., 2022), there is a need for research examining the extent to which these experiences occur during childhood, how they change over the course of typical development, and whether or not they co-occur within the same individuals. Such research would help identify risk factors for misophonia and other sound sensitivities while also shedding light on the development of misophonia.

Focusing on populations of individuals who exhibit characteristics of a disorder but not a diagnosis can be helpful for several reasons. First, misophonic reactions likely occur in the general population to varying extents so understanding this experience is important from a basic science perspective. Second, there is a potentially useful tradition of studying non-diagnosed individuals who exhibit psychiatric symptoms in the broad effort in order to identify endophenotypes (Gottesman and Gould, 2003; Greenwood et al., 2013; Demetriou et al., 2019), i.e., biomarkers that can help reveal the mechanistic source of disorders. The reason this is useful is that only studying those with a diagnosed disorder can lead to ambiguities about whether potential biomarkers are closely tied to the mechanistic basis of the disorder or if instead they are the result of treatments or other consequences of having a chronic disorder. Finally, studying naturalistic coping mechanisms in non-diagnosed individuals who experience mild to moderate misophonic reactions could provide clues about how to treat more severe, diagnosed cases of misophonia. For example, one study (Jager et al., 2020) found that the majority of individuals with misophonia (99%) self-report coping strategies that entail listening to music or producing rhythmic noises that mimic their misophonic triggers, highlighting the need for further research on precisely how music as a stimulus might counter the effects of misophonic reactions. It would therefore be useful to know if musical engagement could promote positive affective auditory experiences that ameliorate some of the problems experienced by those suffering from misophonia, both in terms of overall wellbeing benefits of musical activities but also the specific use of music and music-like stimuli as a coping strategy. Lastly, because misophonic reactions are so common in the general population, further research on these reactions could be informative for basic questions about the development of auditory affective processing more generally.

## Author contributions

All authors have made substantial, conceptual, and intellectual contributions to this manuscript, read, and agreed to the publication of this manuscript.
